# Response to medical students’ perspective: project-based learning approach to increase medical student empathy

**DOI:** 10.1080/10872981.2021.1877100

**Published:** 2021-01-25

**Authors:** Kyong-Jee Kim

**Affiliations:** Department of Medical Education, Dongguk University School of Medicine, 32 Dongguk-ro, Ilsandong-gu, Goyang-si 10326, Korea

**Keywords:** Empathy, teaching and learning methods, project-based learning

I read with great interest, the article by medical students Nathwani and Vedd [1], which was written in response to my article entitled ‘Project-based learning approach to increase medical student empathy’ [2], and I much appreciate their comments on the topic of teaching empathy in medical education. I also agree with the points made by the authors that medical student empathy should be assessed as part of the selection process and that student empathy be allowed to mature through early patient interactions and the teaching of communication skills [1]. Nevertheless, I would like to mention some cultural and curricular issues related to my study.

First, increasing attention is being paid to the development of medical student empathy in Korea, especially from the early phase in medical schools. A national study on Korean medical students concluded they were less empathetic than their Western counterparts [3]. This situation may be the result of Korean culture, which is less dependent on non-verbal communication and regards the portrayal of a calm, unemotional, non-assertive attitude as a virtue [3]. Furthermore, although empathy is highly regarded among Korean doctors, assessments of empathy are not well incorporated in the medical student selection process, which focuses on measurements of cognitive abilities as determined by written tests. This problem is rooted in the Korean education system, which is highly competitive to get into medical school.

Second, the medical students who participated in my study were in the second year of a six-year medical curriculum and had not been exposed to clinical settings or had first-hand experience of patient contact. Furthermore, at the time of the study, it was not feasible to include exposure to learning environments that foster empathic skills through patient contacts in their curriculum, which highlights the need for a formal curriculum in which students are provided authentic learning experiences based on interactions with people in non-clinical settings. Thus, the course designed for our study was intended to enable medical students to develop general empathetic competencies using a project-based learning method, that is an ‘experiential learning’ approach, that might serve as a foundation for a later course on the development of clinical empathy, as I mentioned in my article [2].

Third, although some argue that empathy is innate, research has shown that it can be nurtured by education [4], which supports the need for educational intervention to address the topic. Although I agree with the authors that empathy cannot be nurtured by classroom teaching alone, published reports show that effective instructional approaches can enhance empathy among medical students [5], as demonstrated by the positive and negative influences of classroom and clinical teaching factors and hidden curriculum learning environments on medical student empathy [5]. I hope this exchange contributes to further research of learning environments that might enhance medical student empathy.
